# Distinct transcriptional responses of mouse sensory neurons in models of human chronic pain conditions

**DOI:** 10.12688/wellcomeopenres.14641.1

**Published:** 2018-06-25

**Authors:** M.A. Bangash, Sascha R.A. Alles, Sonia Santana-Varela, Queensta Millet, Shafaq Sikandar, Larissa de Clauser, Freija ter Heegde, Abdella M. Habib, Vanessa Pereira, Jane E. Sexton, Edward C. Emery, Shengnan Li, Ana P. Luiz, Janka Erdos, Samuel J. Gossage, Jing Zhao, James J. Cox, John N. Wood

**Affiliations:** 1 Molecular Nociception Group, Wolfson Institute for Biomedical Research, University College London, London, WC1E 6BT, UK; 2 Comparative Biomedical Science, Skeletal Biology Group, Royal Veterinary College, London, NW1 0TU, UK; 3 College of Medicine, Member of Qatar Health Cluster, Qatar University, Doha, Qatar

**Keywords:** Chronic pain, mouse models, dorsal root ganglia, sensory neurons, microarrays, gene expression

## Abstract

**Background: **Sensory neurons play an essential role in almost all pain conditions, and have recently been classified into distinct subsets on the basis of their transcriptomes. Here we have analysed alterations in dorsal root ganglia (DRG) gene expression using microarrays in mouse models related to human chronic pain.

**Methods: **Six different pain models were studied in male C57BL/6J mice: (1) bone cancer pain using cancer cell injection in the intramedullary space of the femur; (2) neuropathic pain using partial sciatic nerve ligation; (3) osteoarthritis pain using mechanical joint loading; (4) chemotherapy-induced pain with oxaliplatin; (5) chronic muscle pain using hyperalgesic priming; and (6) inflammatory pain using intraplantar complete Freund’s adjuvant. Microarray analyses were performed using RNA isolated from dorsal root ganglia and compared to sham/vehicle treated controls.

**Results: **Differentially expressed genes (DEGs) were identified. Known and previously unreported genes were found to be dysregulated in each pain model. The transcriptomic profiles for each model were compared and expression profiles of DEGs within subsets of DRG neuronal populations were analysed to determine whether specific neuronal subsets could be linked to each of the pain models.

**Conclusions: **Each pain model exhibits a unique set of altered transcripts implying distinct cellular responses to different painful stimuli. No simple direct link between genetically distinct sets of neurons and particular pain models could be discerned.

## Introduction

Chronic pain is a major clinical problem affecting roughly 20% of the population with more than 6% suffering debilitating levels of pain ^1, 2^. Despite the huge clinical burden, little progress has been made in developing more effective analgesic agents. Dorsal root ganglion (DRG) sensory neurons are particularly exciting targets for drug development because of their essential role in driving pain sensations in the central nervous system. With the wider availability of high-throughput RNA-seq, there has been a major effort to define DRG transcriptomes, both at the level of whole ganglia ^3^ and single neurons ^4, 5^. This has allowed a new classification of sets of sensory neurons based on genetic identity, rather than on the rate of action potential propagation (A fibres and C fibres). As well as neurons, non-neuronal cells such as glia ^6^ and immune system cells are found within sensory ganglia, with leukocytes alone making up 5–10% of cells in DRG ^7, 8^.

We wondered if the newly defined sensory neuron subsets were differentially activated in different pain models. We therefore performed microarray analyses of RNAs isolated from the DRG of male mice subjected to six different interventions that mimic common chronic pain in human patients. DRG were dissected after the development of peak pain behaviours in each case. The six different chronic pain models were: cancer cell injection in the intramedullary space of the femur as a model for bone cancer pain; partial sciatic nerve ligation (PSL) for neuropathic pain; mechanical joint loading for osteoarthritis pain; oxaliplatin-induced painful neuropathy for chemotherapy-induced pain; hyperalgesic priming model for chronic muscle pain and intraplantar complete Freund’s adjuvant (CFA) for inflammatory pain. The rodent models for PSL ^9^, CFA ^10^, chemotherapy induced pain with oxaliplatin ^11^, bone cancer pain ^12^ and hyperalgesic priming ^13– 15^ are well characterized. The mechanical joint loading model robustly recapitulates behavioural signs and symptoms of osteoarthritis ^16^.

 By comparing altered levels of gene expression in six distinct chronic pain models we hoped to gain insights into the neuronal subsets that are particularly important in different pain conditions. We also could determine genes and related pathways that are common or unique to each condition. Here we show that there is no simple relationship between transcriptional changes in the newly defined subsets of sensory neurons and particular painful insults. However, we have identified several known and previously unreported genes altered in expression in each pain condition. Relevant pathways and upstream regulators were also analysed using Ingenuity Pathway Analysis including cytokines, transcription factors (TFs), G-protein coupled receptors (GCPRs) and ion channels. The results from these experiments highlight the complexity of sensory neuron cell types involved in nociceptive responses. Although altered DRG gene expression may not necessarily play a causal role in pain, some dysregulated genes are potential analgesic drug targets.

## Methods

All experiments were performed in accordance with the UK Animals (Scientific Procedures) Act 1986 with prior approval under a Home Office project licence (PPL 70/7382). Mice were kept on a 12-h light/dark cycle and provided with food and water *ad libitum.* All animals were acclimatized for 1 week to the facility before the start of the experiment. Mice were housed in individually ventilated cages (Techniplast GM500 Mouse IVC Green line) containing Lignocel bedding with a maximum of 5 adult mice per cage. All tests and surgeries were conducted using adult male C57BL/6J mice supplied by Charles River and Envigo (specific numbers detailed in the figure legends and ages detailed below for each model). Sample size for each behavioural model was calculated using G*Power (Ver. 3.1.9.2) for a power of 0.8 ^17^. Surgical procedures were performed by trained researchers and under aseptic conditions. All efforts were made to ameliorate any suffering of the animals with surgery performed under anaesthesia. Mice were euthanized by CO _2_ asphyxiation followed by cervical dislocation.

### Cancer-induced bone pain

LL/2 Lewis Lung carcinoma cells (ATCC) were cultured in DMEM supplemented with 10% FBS and 100 units/ml Penicillin/Streptomycin for at least 2 weeks prior to surgery (cell culture reagents supplied by Thermo Fisher). Cells were split at 70–80% confluence four days prior to surgery. On the day of surgery, cells were harvested with 0.05% Trypsin-EDTA solution and resuspended in 1X DMEM at a final concentration of 2×10 ^7^ cells/ml and kept on ice till used. Viability of Lewis lung carcinoma cells was confirmed at the end of surgery, showing 26% dead cells compared to 11% before surgery. The cancer cells were introduced to 12 week old mice, as previously described ^18, 19^. Sham-operated control mice underwent the same surgery, but were inoculated with DMEM medium alone. Ipsilateral L2-L4 DRGs from test and paired sham animals were collected when a limb score of 2 was measured in the test animals (see section *Behavioural tests*).

### microCT (uCT) for bone cancer

Femurs were post-fixed in 4% PFA (Sigma-Aldrich) for 24h and kept in 70% ethanol (VWR) till scanning with microCT. Images were acquired with Skyscan software at 6.41um/pixel with 0.6 degrees rotation steps and 2 frame averaging. Image data was reconstructed with NRecon software. A CT-analyzer was used to select a 1mm volume of interest (VOI) region, starting at 0.6mm from the growth plate. Bone mineral density was determined by plotting attenuation coefficients against a standard curve determined with two phantoms with known density. Representative images were binarized with ImageJ (1.52c) and 3D Viewer extension (Java 3D 1.6 with 3D Viewer plugin 4.0.2) used for 3D reconstructions of femur scans.

### Partial sciatic nerve ligation (PSL) model

Surgical procedures were performed under isoflurane anaesthesia (2–3%). A partial nerve injury in 14 week old mice was induced by tying a tight ligature with 6-0 silk suture around approximately 1/3 to 1/2 the diameter of the sciatic nerve, similar to the approach described in rats ^9^. Ipsilateral and contralateral L3-L5 DRGs were dissected prior to RNA isolation on post-surgery day 16.

### Mechanical joint loading (MJL) model

*In vivo* loading: Osteoarthritis was induced by subjecting 12 week old mice to a two week loading regimen using an electronic materials testing machine (Bose 3100). Throughout the loading episodes, mice were anaesthetised with isoflurane 3.5% (±0.5%) with the right tibia positioned vertically between two custom-made cups which fixate the knee and ankle joint in deep flexion ^20^. The loading regimen was the same as previously described ^16^. Loading was repeated three times per week for two consecutive weeks. The non-loaded control group consisted of age- and cage-matched mice that were not subjected to a loading regimen but received isoflurane anaesthesia for the same duration as loaded mice. Ipsilateral L3–L5 DRGs were dissected from loaded and non-loaded mice after three weeks.

### Oxaliplatin pain model

Seven week old mice were injected with either oxaliplatin (6mg/kg/i.p., Sigma-Aldrich) or vehicle solution (5% glucose, Sigma-Aldrich) as a control twice weekly for four weeks ^11, 21^. Ipsilateral L3–L5 DRGs were dissected prior to RNA isolation from oxaliplatin and vehicle treated mice on day 28.

### Chronic muscle pain model

Eight week old mice were injected twice, four days apart, with 30ul of 3% carrageenan (Sigma-Aldrich) (primed group; n=4) or saline (VWR) (control group; n=4) into the ipsilateral right gastrocnemius muscle ^13^. Ipsilateral L3–L5 DRGs were dissected prior to RNA isolation on day 29.

### Complete Freund’s adjuvant (CFA) pain model

Eleven week old mice received intraplantar injection of 20 ul of Complete Freund’s adjuvant (CFA, Sigma-Aldrich) or 0.9% sodium chloride solution (VWR) into the left hind paw ^22, 23^. Ipsilateral L3-L5 DRGs were then dissected prior to RNA isolation from CFA and saline treated mice 2 days after treatment.

### Behavioural tests

For behavioural experiments, animals were acclimatized to the equipment for at least 2 days prior to testing. Observers who performed behavioural experiments were blind to the test/sham groups. The cancer induced bone pain model used a limb score assessment, as previously described ^19^. A limb score of 2 (i.e. significant limping) was used as the checkpoint for ending the experiment with DRGs and ipsilateral femurs collected.

Mechanical sensitivity was measured using the up-down method ^24^ and static weight bearing ^18^ assays. Thermal nociceptive thresholds were determined by measuring paw withdrawal latency using the Hargreaves’ apparatus ^25^. The response to noxious cold was measured using the cold plantar assay ^26^.

### RNA extraction and microarrays

RNA was extracted using TRIzol Reagent (Life Technologies) and Purelink RNA micro kit (Thermo Fisher) according to the manufacturer’s instructions. RNA samples from four animals displaying the most marked pain behaviours for each model were sent for microarray analyses together with four controls (Eurofins AROS) using the Affymetrix GeneChip Mouse Transcriptome Array 1.0 and WT pico kit. Four replicates give a statistical power of >0.8 to detect a 2 fold change ^27, 28^. Microarray data has been deposited at Gene Expression Omnibus Array Express for public use with reference number E-MTAB-6864.

### Differential gene expression (DGE) and splice variant analysis

Differential gene expression (DGE) and splice variant analysis of transcriptome array data was performed using Transcriptome Analysis Console (TAC Ver 4.0, Thermo Fisher). Normalization was carried out according to the manufacturer’s default protocol converting probe cell intensity (CEL files) into signal data (CHP files). An ANOVA (eBayes) cut-off p-value of <0.05 was used to identify DEGs, with the fold change filter then used to sort genes. Genes without a curated gene symbol associated with the Affymetrix probe set were excluded from downstream DGE analyses. Differentially expressed genes (DEGs) were correlated with known expression in DRG neuronal subtypes ^4^. Splice variant analysis was performed using the Gene + Exon function in TAC which uses a gene normalized intensity value (ratio of probe set intensity to gene expression level). The cut off was set at Splicing Index of 2, splicing ANOVA with p<0.05, gene expressed in both conditions, probe selection region (PSR)/ junction expressed in at least one condition and gene containing at least one PSR. Non-coding genes were excluded from the splicing analyses. An exon event score indicated consensus alternative splicing event score where a score of 1 represents a high likelihood of splicing event. Heatmaps, volcano plots and statistical analyses were generated using GraphPad Prism 7.

### Ingenuity Pathway Analysis (IPA)

Ingenuity Pathway Analysis (IPA, Qiagen) for upstream regulators was performed according to the manufacturer’s instructions. IPA primarily utilizes published results of knockdown or knockout studies of genes characterized as growth factor, kinase/phosphatase, transcription/translation regulator, cytokine, transmembrane receptor, enzyme, ion channel, transporter, G-protein coupled receptor, peptidase, and microRNA. This analysis examines target genes from the array dataset, compares their direction of change to what is expected from the literature to predict likely upstream regulators using overlap p-value (Fishers Exact Test, p<0.01) and an activation z-score corrected for bias. The top upstream regulators with an activation z-score were examined for each pain pathway. The activation z-score was used to make a prediction regarding activation or inhibition of the upstream regulator.

## Results

We modelled six different chronic pain conditions in mice and used behavioural assays to confirm the development of mechanical or thermal hypersensitivity, followed by transcriptomic analyses of whole dorsal root ganglia tissue. Significant DGE between naïve and injured conditions and alternative splicing was assessed for each model. DGE values for every gene on the mouse transcriptome array and alternative splicing changes are available in Supplementary Table 1.

### Cancer-induced bone pain

**Figure 1.**
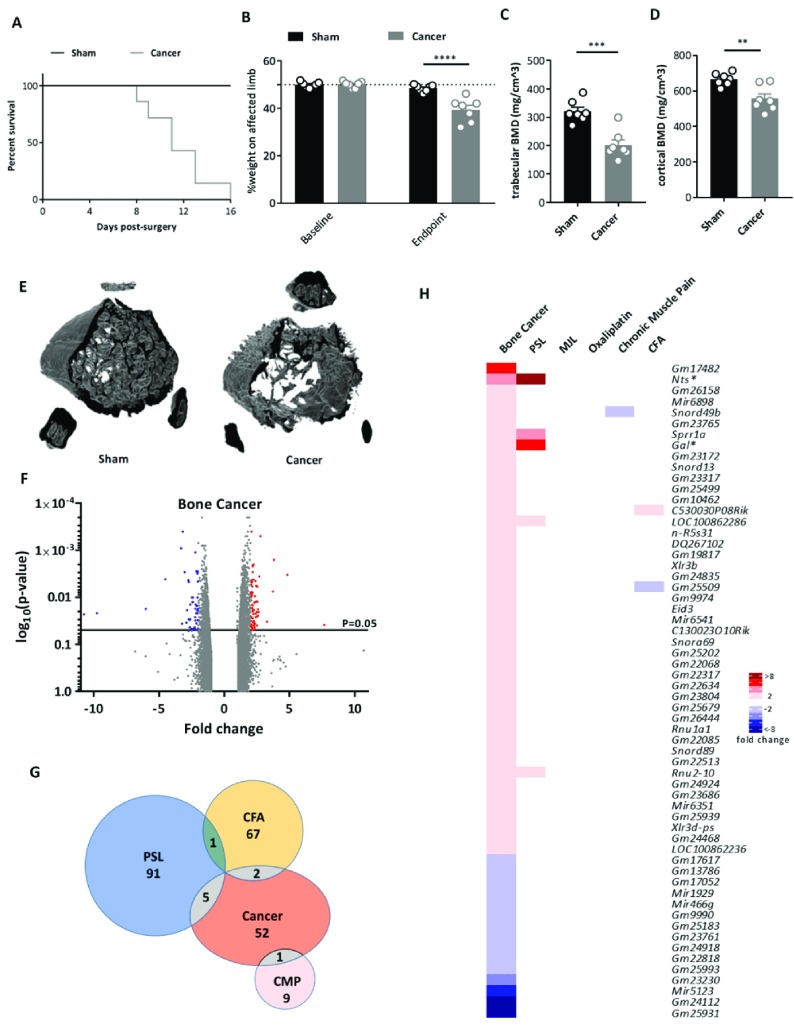
Differential gene expression in the cancer induced bone pain model. ( **A**) Survival curve after surgery for sham (black line, n=7) and cancer (grey line, n=7) animals with endpoint defined as clear limping on the affected limb (limb score=2). Log-rank test: p=0.0056. ( **B**) Percentage weight bearing on the affected limb is significantly reduced at the endpoint (limb score=2) for cancer compared to sham animals (2-way ANOVA with posthoc Bonferonni test: p<0.0001.) ( **C**) Trabecular bone mineral density (unpaired t-test: p=0.0003) and ( **D**) cortical bone mineral density (unpaired t-test: p=0.0033) in cancer vs. sham animals. ( **E**) Representative 3D reconstructions of a 1mm VOI at 0.6mm from growth plate from uCT data (7 replicates per group). ( **F**) Volcano plot for all genes identified in the bone cancer pain array. Each dot represents a single gene (red=upregulated; blue=downregulated). ( **G**) Venn diagram showing the number of differentially expressed DRG genes (fold change >2, <-2, p<0.05) shared with other pain models. ( **H**) Heat map of upregulated (red) and downregulated (blue) DRG genes (fold change >2, <-2, p<0.05) and a comparison of shared genes across models. Genes with an asterisk are present within the *Pain Genes Database* of pain-related transgenic knockout studies ^34^.

Carcinoma cells were introduced into the mouse femur and pain behaviour measured daily to determine the limb use score (4: normal use of limb, 3: slight limping, 2: clear limping; see *Methods*). Mice were culled when a limb score of 2 was reached, which ranged from Day 8–16 post surgery ( Figure 1A). Weight bearing tests confirmed a significant reduction in use of the affected limb ( Figure 1B). Bone mineral density (BMD) was evaluated as an additional indicator of the cancer phenotype and was shown to be significantly decreased in cancer mice compared to sham controls ( Figure 1C, D). This is also shown in Figure 1E using a micro-CT reconstruction.

Dorsal root ganglia (ipsilateral L2–L4) were isolated from test mice and paired sham controls and RNA expression levels analysed using microarrays. The volcano plot ( Figure 1F) shows that all significantly dysregulated genes (p<0.05) were differentially expressed between ±10-fold with only 1 non-coding gene ( *Gm25931*) falling outside of this range (fold change of -10.75). Interestingly, very few of the differentially expressed DRG genes in the cancer induced bone pain model were shared with other models ( Figure 1G). A heat map was generated for genes that were significantly up or down regulated by 2 fold or more ( Figure 1H). Forty-five genes were found to be upregulated >2 fold and 15 genes downregulated. Dysregulated genes were coding and non-coding, with many genes annotated as ‘predicted’ ( Supplementary Table 1). Excluding these ‘predicted’ genes, the top 5 most upregulated genes were *Nts*, *miR6898*, *Snord49b*, *Sprr1a* and *Gal* ( Figure 1H). The cancer induced bone pain model induced significant changes in 23 microRNAs in the >1.5 fold range, indicating the potential role of miRNAs in cancer pain modulation ( Supplementary Figure 2A).

### Partial sciatic nerve ligation (PSL) model

**Figure 2.**
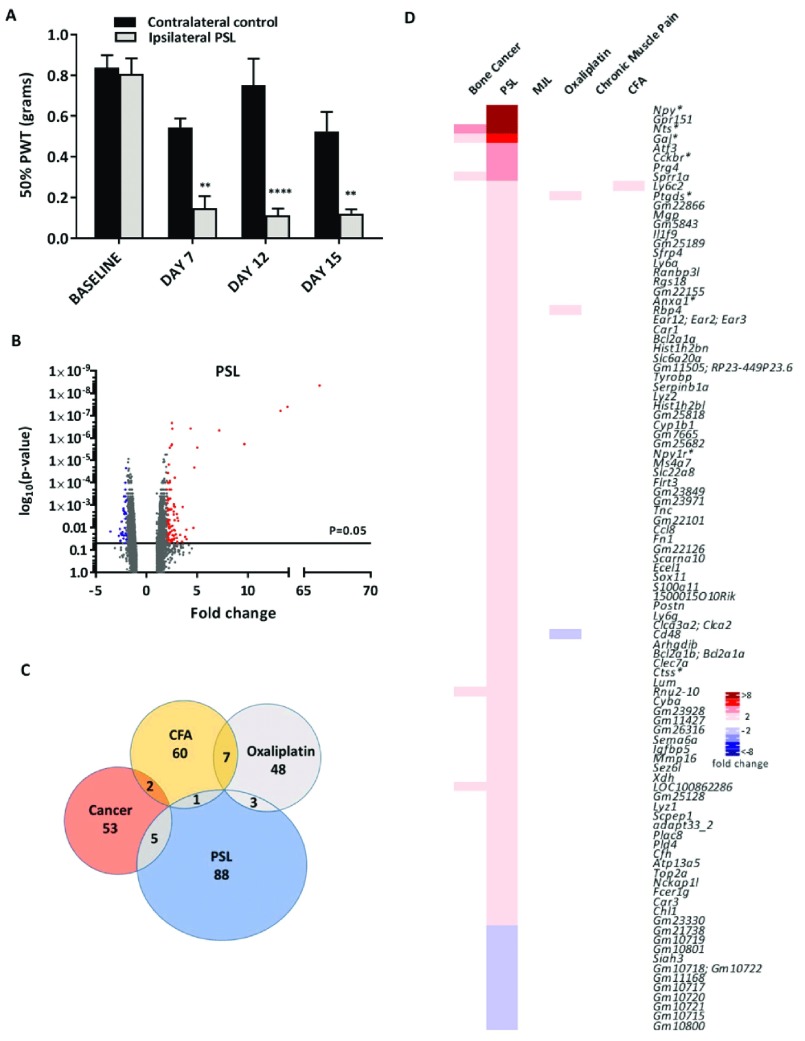
Differential gene expression in the partial sciatic nerve ligation (PSL) neuropathic pain model. ( **A**) Mechanical sensitivity using von Frey filaments on ipsilateral and contralateral paws following PSL surgery (n=4, **indicates p<0.01, ****indicates p<0.0001, 2-way ANOVA with posthoc Bonferonni test). PWT = paw withdrawal threshold. ( **B**) Volcano plot for all genes identified in the PSL pain model array. Each dot represents a single gene (red=upregulated; blue=downregulated). ( **C**) Venn diagram showing the number of differentially expressed DRG genes (fold change >2, <-2, p<0.05) shared with other pain models. ( **D**) Heat map of upregulated (red) and downregulated (blue) DRG genes (fold change >2, <-2, p<0.05) and a comparison of shared genes across models. Genes with an asterisk are present within the *Pain Genes Database* of pain-related transgenic knockout studies ^34^.

In the PSL neuropathic pain model mice developed mechanical allodynia from Day 7 post surgery, as characterized by a significant reduction in the 50% paw withdrawal threshold (PWT) in the injured (ipsilateral) compared to the non-injured (contralateral) paw ( Figure 2A). Pain persisted for 15 days post-surgery, with ipsilateral and contralateral L3-L5 DRGs isolated on day 16 and RNA analysed by microarrays.

The volcano plot ( Figure 2B) shows that all significantly dysregulated genes (p<0.05) were differentially expressed between ±10-fold with the exception of *Nts*, *Gpr151* and *Npy* (respective fold changes of 13.21, 13.88 and 66.18). Similar to the cancer model, very few PSL stimulated genes were shared with other pain models ( Figure 2C). The PSL model stimulated changes in the largest cohort of DRG genes in the 2 fold change range (97 genes) compared to the other 5 models ( Figure 2D). The top 5 most upregulated genes, *Npy*, *Gpr151*, *Nts*, *Gal* and *Atf3*, have all been previously reported as being upregulated in neuropathic pain ^29– 32^. Eighty-six genes were identified as being upregulated more than 2 fold with several of these being non-coding RNAs ( Supplementary Table 1). Fourteen microRNAs were upregulated >1.5-fold and 5 were downregulated <-1.5- fold (p<0.05, ANOVA) ( Supplementary Figure 2B).

### Mechanical joint loading (MJL) model

**Figure 3.**
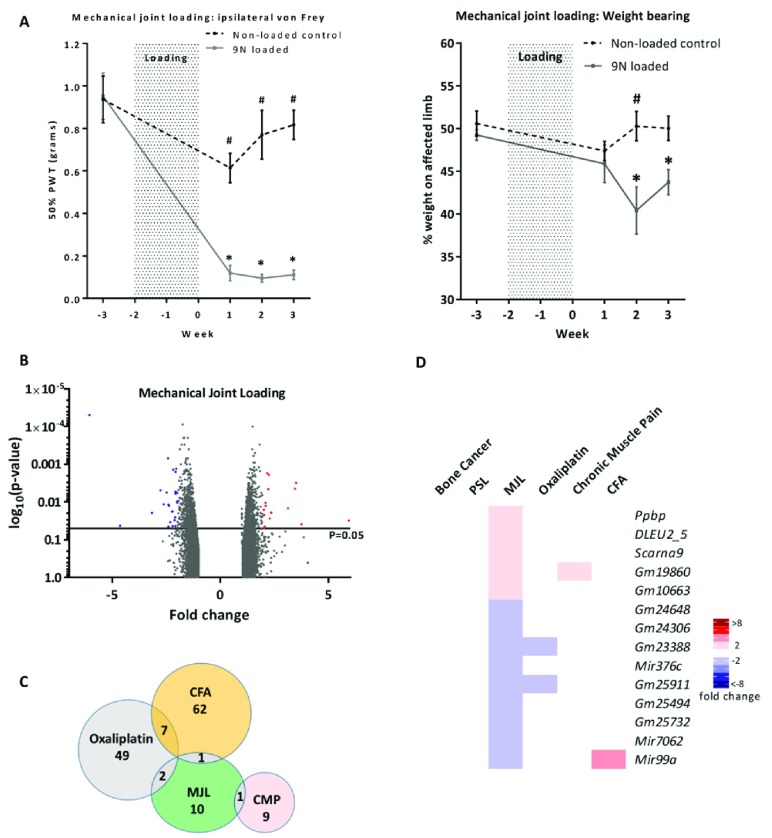
Differential gene expression in the mechanical joint loading (MJL) model. ( **A**) Development of mechanical hypersensitivity and altered weight bearing after osteoarthritis (OA) induction by mechanical joint loading. Mice were loaded three times per week for two weeks at 9N (grey line, error bars given as SEM, n=6) in order to induce OA. Behavioural measurements were taken before OA induction and each week for three weeks post loading. These values were compared to a non-loaded isoflurane control (black hashed line, error bars given as SEM, n=6). Significant changes between non-loaded animals and loaded animals are indicated with a # (p<0.05, 2-way ANOVA with posthoc Dunnett or Sidak tests) whilst significant changes within experimental groups over time (compared to baseline value) are indicated with a * (p<0.05, 2-way ANOVA with posthoc Dunnett or Sidak tests). ( **B**) Volcano plot for all genes identified in the MJL array. Each dot represents a single gene (red=upregulated; blue=downregulated). ( **C**) Venn diagram showing the number of differentially expressed DRG genes (fold change >2, <-2, p<0.05) shared with other pain models. ( **D**) Heat map of upregulated (red) and downregulated (blue) DRG genes (fold change >2, <-2, p<0.05) and a comparison of shared genes across models.

Osteoarthritis was induced through a two-week mechanical joint loading protocol after which animals developed significant mechanical hypersensitivity and altered weight bearing ( Figure 3A). Ipsilateral L3–L5 DRGs from loaded and non-loaded mice were extracted 3 weeks after the loading regimen, RNA extracted and analysed for DEGs. The volcano plot ( Figure 3B) shows that all significantly dysregulated genes (p<0.05) were differentially expressed between ±7-fold. Only fourteen genes were dysregulated >2 fold ( Figure 3D) with only 1–2 of these genes also differentially expressed in the other pain models ( Figure 3C). The top three upregulated genes ( *Ppbp*, *DLEU2_5* and *Scarna9*) have no previous association with osteoarthritic pain.

### Oxaliplatin-induced painful neuropathy

**Figure 4.**
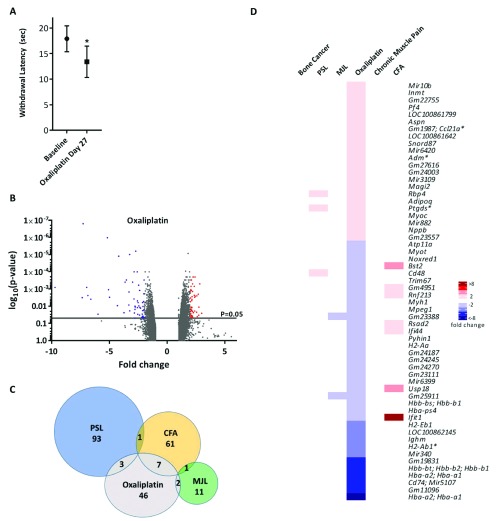
Differential gene expression in the oxaliplatin pain model. ( **A**) Cold hypersensitivity induced by oxaliplatin (6 mg/kg, i.p.) at Day 27 (5% glucose solution was used as control). Significant changes are indicated with * (n=6 per group, *p<0.05, paired t-test). ( **B**) Volcano plot for all genes identified in the oxaliplatin model array. Each dot represents a single gene (red=upregulated; blue=downregulated). ( **C**) Venn diagram showing the number of differentially expressed DRG genes (fold change >2, <-2, p<0.05) shared with other pain models. ( **D**) Heat map of upregulated (red) and downregulated (blue) DRG genes (fold change >2, <-2, p<0.05) and a comparison of shared genes across models. Genes with an asterisk are present within the *Pain Genes Database* of pain-related transgenic knockout studies ^34^.

Oxaliplatin is a chemotherapeutic drug known to induce mechanical and cold allodynia ^33^. Mice exposed to oxaliplatin were found to have a reduced cold pain threshold in the cold plantar test ( Figure 4A). Twenty-eight days following the first oxaliplatin treatment, ipsilateral L3–L5 DRGs were extracted from test and vehicle treated mice and analysed for differential gene expression. The volcano plot ( Figure 4B) shows that all significantly dysregulated genes (p<0.05) were differentially expressed between ±10-fold. Oxaliplatin treatment resulted in 58 dysregulated genes in the >2 fold range, which in contrast to other models, the majority (36 genes) being down- rather than up-regulated ( Figure 4D). Out of the 58 DEGs, 7 were shared with the CFA model, 3 with the PSL model and 2 with the mechanical joint loading model ( Figure 4C).

### Chronic muscle pain (CMP)

**Figure 5.**
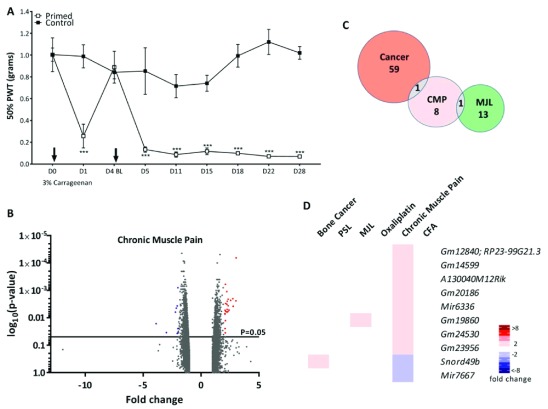
Differential gene expression in the chronic muscle pain model. ( **A**) Widespread mechanical hypersensitivity in a mouse model of the transition from acute to chronic musculoskeletal pain. Mice were injected twice with 3% carrageenan i.m. (primed group) or saline (control group) into the gastrocnemius muscle (n=4 per group). Injections are indicated by arrows and mechanical withdrawal thresholds were assessed in the glabrous skin of the ipsilateral hind paw. Significant changes denoted by *** (***p<0.001 Primed vs Control, 2-way ANOVA with repeated measures and Bonferroni post hoc tests). ( **B**) Volcano plot for all genes identified in the chronic muscle pain model array. Each dot represents a single gene (red=upregulated; blue=downregulated). ( **C**) Venn diagram showing the number of differentially expressed DRG genes (fold change >2, <-2, p<0.05) shared with other pain models. ( **D**) Heat map of upregulated (red) and downregulated (blue) DRG genes (fold change >2, <-2, p<0.05) and a comparison of shared genes across models.

A hyperalgesic priming model was used to study chronic muscle pain, whereby the gastrocnemius muscle was injected twice, four days apart, with 3% carrageenan. Prolonged mechanical hypersensitivity resulted from the hyperalgesic priming ( Figure 5A). On Day 29, ipsilateral L3-L5 DRGs were extracted from test and vehicle treated mice and analysed for DGE. The volcano plot ( Figure 5B) shows that all significantly dysregulated genes (p<0.05) were differentially expressed between ±5-fold. Interestingly, only ten genes were dysregulated >2 fold ( Figure 5D) with *Snord49b* shared with the cancer-induced bone pain model and *Gm19860* shared with the mechanical joint loading model ( Figure 5C).

### CFA inflammatory pain model

**Figure 6.**
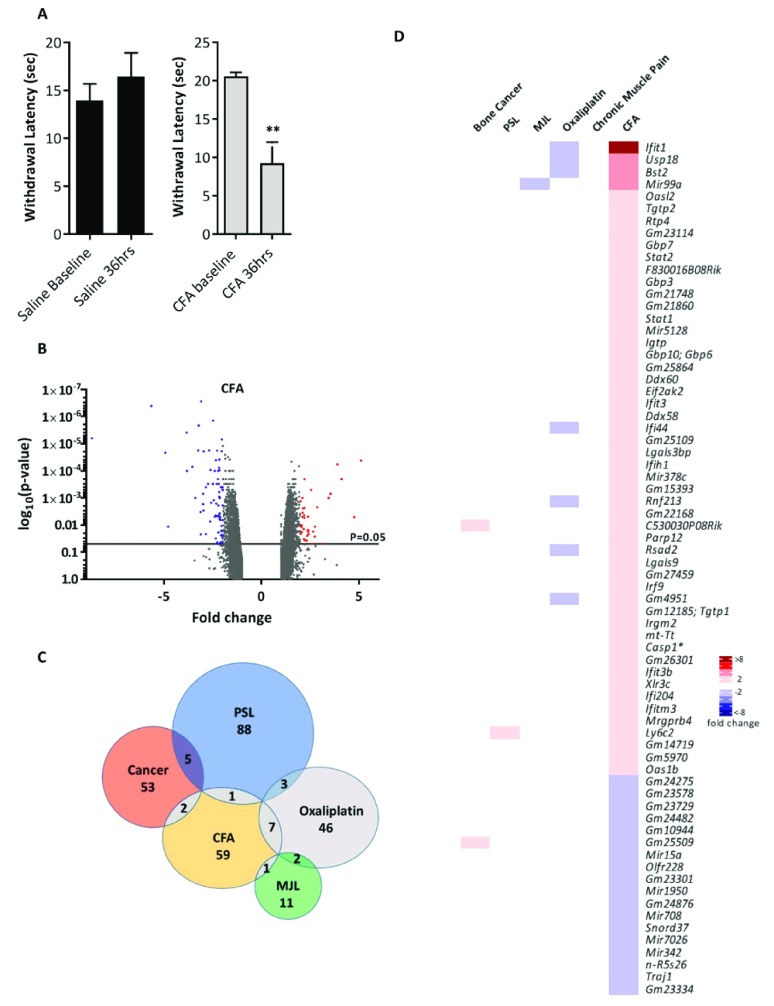
Differential gene expression in the complete Freund’s adjuvant (CFA) inflammatory pain model. ( **A**) Heat hypersensitivity post CFA intraplantar injection (20 microliters) in mice. Significant changes are indicated with ** (n=6 per group, ** p<0.01, 2-way ANOVA). ( **B**) Volcano plot for all genes identified in the CFA pain model array. Each dot represents a single gene (red=upregulated; blue=downregulated). ( **C**) Venn diagram showing the number of differentially expressed DRG genes (fold change >2, <-2, p<0.05) shared with other pain models. ( **D**) Heat map of upregulated (red) and downregulated (blue) DRG genes (fold change >2, <-2, p<0.05) and a comparison of shared genes across models. Genes with an asterisk are present within the *Pain Genes Database* of pain-related transgenic knockout studies ^34^.

Intraplantar injection of CFA was used to induce a robust inflammatory pain phenotype, with significant thermal hypersensitivity observed thirty-six hours post CFA injection ( Figure 6A). Ipsilateral L3–L5 DRGs were extracted 2 days post CFA and RNA analysed for differential gene expression. The volcano plot ( Figure 6B) shows that all significantly dysregulated genes (p<0.05) were differentially expressed between ±9-fold. Seventy genes were differentially expressed >2 fold with the majority of genes (52) being upregulated ( Figure 6D). Once again, very few dysregulated genes were shared between CFA and the other models ( Figure 6C).

### Expression of pain-related DEGs in DRG neuronal subsets

**Figure 7.**
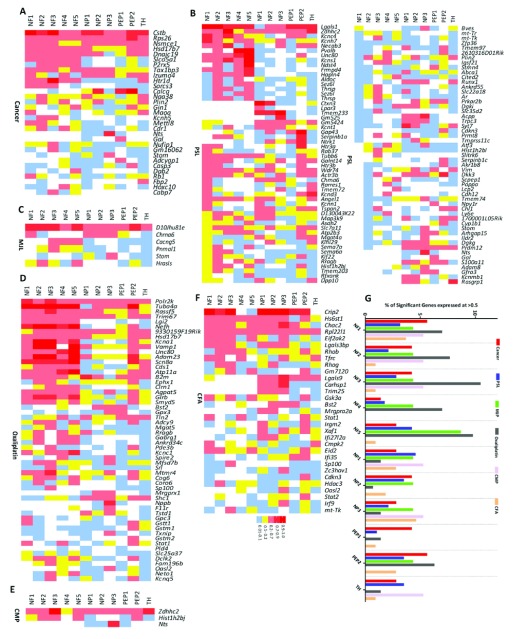
Pattern of dorsal root ganglion (DRG) neuron expression of differentially expressed genes (DEGs) identified in the six pain models. Expression pattern in ( **A**) Bone cancer, ( **B**) partial sciatic nerve ligation (PSL), ( **C**) mechanical joint loading (MJL), ( **D**) Oxaliplatin, ( **E**) chronic muscle pain (CMP) and ( **F**) complete Freund’s adjuvant (CFA) models of the DEGs and their basal expression pattern in the 11 different subtypes of DRG neurons (NF1-5, NP1-3, PEP1-2, TH) based on the classification described by Usoskin *et al*. ^4^. NF1, NF2 and NF3 correspond to low-threshold mechanoreceptors (LTMRs); NF4 and NF5 correspond to proprioceptors; NP1, NP2 and NP3 are unmyelinated, nonpeptidergic neurons; PEP1 are unmyelinated, peptidergic neurons; PEP2 are myelinated, peptidergic neurons; and TH neurons are type C low-threshold mechanoreceptors (C-LTMRs) that are unmyelinated. Analysis has been restricted to DEGs with a fold-change greater than 1.5 or less than -1.5 (p<0.05) with at least 20% expression in 1 or more of the neuronal subgroups in the Usoskin *et al.* dataset ^4^. ( **G**) Transcriptional preference for distinct DRG neuronal subpopulations (described previously) in each pain model. This graphical estimation is based on a particular gene being expressed in 50% or more of a particular neuronal subpopulation and presented as a percentage of the total DEGs shared with the Usoskin *et al.* dataset ^4^ at >1.5 or <-1.5 fold (p<0.05).

Single cell RNA sequencing of mouse DRG neurons has identified 11 distinct subsets of neurons ^4^. To determine whether particular DRG neuronal populations are enriched with DEGs, we created heat maps for each model that show expression of every neuronal DEG (fold change >±1.5 fold; p<0.05) and compared this with the basal expression of each gene in the Usoskin *et al*. dataset ^4^ ( Figure 7A–F, Supplementary Figure 1A–F). Next, the percentage of DEGs was correlated with each subpopulation of DRG neurons ( Figure 7G). This indicated that there were no clear trends for the expression pattern of the DEGs for each model, with the exception of oxaliplatin where DEGs clustered in the NF1-5 and PEP2 subgroups (myelinated neurofilament-heavy ( *Nefh*) positive neurons).

### Analysis of upstream regulators of neuronal DEGs in chronic pain models

**Figure 8.**
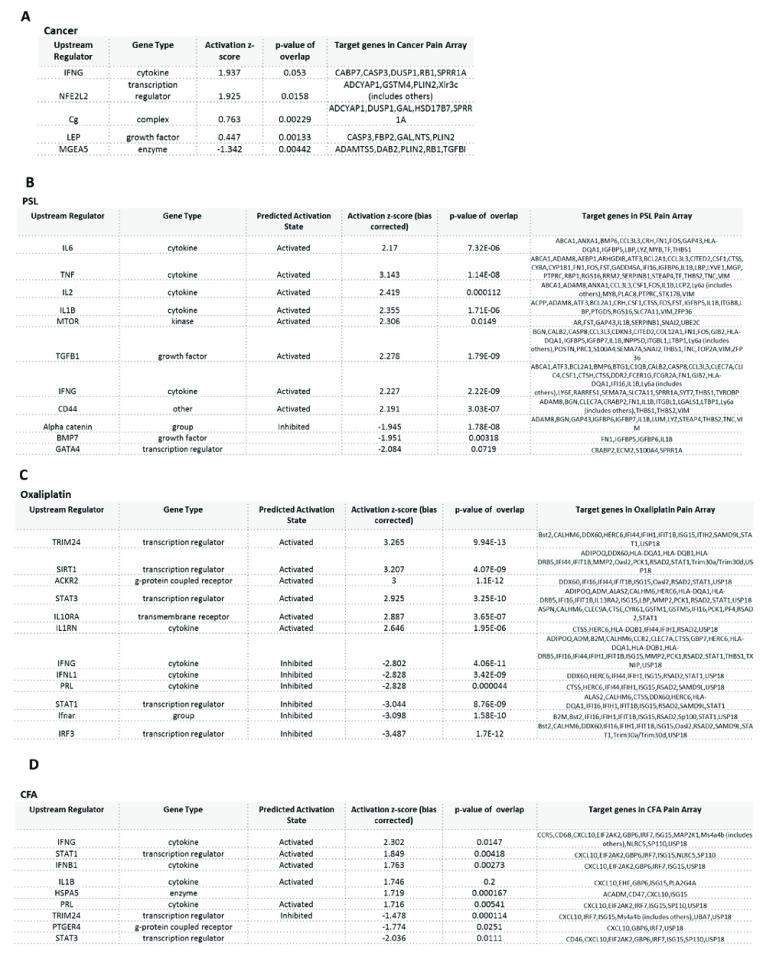
Top upstream regulators of differentially expressed genes identified in bone cancer, partial sciatic nerve ligation (PSL), oxaliplatin, and complete Freund’s adjuvant (CFA) models. Upstream regulators were identified for ( **A**) Bone cancer, ( **B**) PSL, ( **C**) Oxaliplatin and ( **D**) CFA. Bias corrected activation z-score predicts likelihood of activation (+ive score) or inhibition (-ive score) of the molecular pathway.

Next, we analysed pathways and upstream regulators implicated in the four pain conditions with the highest number of DEGs using the Ingenuity Pathway Analysis program. The top upstream regulators in the bone cancer pain model are shown in Figure 8A (p-value of overlap <0.05, ANOVA) and includes cytokine IFNG (activation z-score 1.9); growth factor LEP (activation z-score 0.4); and transcription regulator NFE2L2 (activation z-score 1.9). The analysis was unable to determine whether these regulators were activated or inhibited. The top upstream regulators in the PSL model are depicted in Figure 8B (p-value of overlap<0.05, ANOVA), and includes growth factors TGFB1 (activation z-score 2.2, predicted to be activated) and BMP7 (activation z-score -1.9); cytokines TNF, IL-6, IL-1-beta (activation z-scores > 2, all predicted to be activated in PSL); kinase MTOR (activation z-score 2.3, predicted to be activated); and the transcription regulator GATA4 (activation z-score -2.8). In the oxaliplatin model ( Figure 8C) the top upstream regulators (p<0.05, ANOVA) involved included cytokines IFNG, IFNL1, PRL (activation z scores< -2, all predicted to be inhibited) and IL1RN (activation z score >2, predicted to be activated); G-protein coupled receptor ACKR2 (activation z score 3, predicted to be activated); and transcription regulators TRIM24, SIRT1, STAT3 (activation z scores > 2, all predicted to be activated) and STAT1 (activation z score <-3, predicted to be inhibited). Finally, for CFA the top upstream regulators (p<0.05, ANOVA) included cytokines IFNG, IL-1B and IFN-B1 (activation z scores>1.7, all predicted to be activated); transcription regulator STAT1 (activation z score 1.8, predicted to be activated) and TRIM24 (activation z score -1.47, predicted to be inhibited) ( Figure 8D). We observed interesting patterns of activation compared to inhibition of upstream regulators between pain models. For example, STAT1 is activated in oxaliplatin, but inhibited in the CFA model; IFNG is activated in CFA and PSL, but inhibited in the oxaliplatin model.

## Discussion

DRG sensory neurons are a heterogeneous population that have recently been classified according to their gene expression profiles ^4^. By modelling six chronic pain conditions in mice we aimed to (1) identify dysregulated DRG genes in each model; (2) map dysregulated genes to one or more of the 11 neuronal subsets; and (3) determine whether different pain models shared similar dysregulated genes and/or neuronal subsets. Overall our analysis highlighted how dissimilar the different models were in respect to their transcriptomic profiles with the majority of dysregulated genes not shared between models (see Venn diagrams in Figure 1– Figure 6). This implies that chronic pain can arise from a diverse set of mechanisms involving different genes and pathways. Although transcriptional changes may not be causative in the pain induction, the present study suggests distinct cell and molecular mechanisms are involved with associated transcriptional correlates in the pain models studied. Although some key genes were shared between models, such as Neurotensin and Galanin being upregulated in cancer induced bone pain and PSL neuropathic pain models, the majority of dysregulated genes were exclusive to each pain model.

We also largely failed to link narrow subsets of DRG neurons to particular chronic pain models using microarray analyses (see Figure 7). Our aim was to identify dysregulated genes from the microarrays and map these genes to the neuronal populations previously outlined using single cell RNAseq data ^4^. However, this approach was limited by the fact that the published RNAseq data is derived from naïve mice (i.e. not in pain) and potentially the gene expression patterns across the neuronal subsets may dynamically change in the pain models. This is certainly true for genes such as Galanin which is markedly upregulated and expressed in ∼40–50% of DRG neurons following axotomy ^32^ and BDNF, which is upregulated in medium and large diameter DRG neurons in neuropathic pain ^35^. Furthermore, genes that have no DRG expression in the Usoskin *et al.* dataset (i.e. zero sequencing reads) were not included in our transcriptomic analyses, but could be expressed following injury. For example, Neuropeptide Y is dramatically upregulated in DRG neurons following peripheral nerve injury, but is not expressed in basal conditions ^29^. Thirdly, many genes are broadly expressed in several DRG populations, meaning that restricted critical subsets are difficult to highlight.

Recent advances in deep sequencing have dramatically increased the number of known transcribed genes ^36^. The Mouse Transcriptome Array that we used gives a comprehensive coverage of the mouse transcriptome, with >214,000 transcripts represented. Genes that are represented include both coding and non-coding genes (such an lncRNAs, small RNAs and expressed pseudogenes). Although such genes are now known to be expressed, gene annotation and understanding of gene function is lagging behind. This is highlighted in our transcriptomic analyses where a high number of predicted genes (typically with the ‘Gm’ prefix) showed differential expression. A major challenge is to determine the function of these types of genes and to ascertain the contribution, if any, to the pain phenotypes observed. Similarly, although the dense probe coverage on the array gives the opportunity to assess differential splicing patterns ( Supplementary Figure 3), experimental verification is needed to prove that the changes in splicing patterns are relevant.

Given the unique sets of underlying genes, we attempted to identify potential common upstream regulators using IPA analyses. We identified several common cytokines as upstream regulators of DEGs in our pain models consistent with the role of cytokines in generation and maintenance of pain ^37, 38^, pointing to the interaction of DRG neurons and immune cells as a key point of focus. T cells are known to infiltrate DRGs in models of neuropathic pain ^39– 41^, however whether the cytokine related downstream DEGs identified in our pain models are being induced by immune cells remains to be investigated.

In summary, we present a systematic study of the transcriptomic changes in six different chronic mouse pain models as a resource for the pain community. Although gene expression changes may not be causally linked to the pain phenotypes observed, the transcriptomic profiles reported here highlight the diversity of cell and molecular mechanisms apparent in different pain conditions. Potential therapeutic gene targets for the distinct chronic pain conditions studied may also be identified after further mechanistic studies.

## Data availability

Microarray data has been deposited at Gene Expression Omnibus Array Express for public use with reference number E-MTAB-6864.

DGE data ( Supplementary Table 1), replicates for Figure 1E (3D reconstruction images) and raw values in a GraphPad Prism file for Figure 1A-D, Figure 2A, Figure 3A, Figure 4A, Figure 5A and Figure 6A for all replicates are deposited in OSF: https://doi.org/10.17605/OSF.IO/DG7Z3 ^42^

Data are available under the terms of the Creative Commons Zero "No rights reserved" data waiver (CC0 1.0 Public domain dedication).

## Supplementary material

**Supplementary Table 1: Differential gene expression values for all genes on the mouse transcriptome array and alternative splicing changes.** Differential gene expression and splice variant analysis of transcriptome array data was performed using Transcriptome Analysis Console (TAC Ver 4.0, Thermo Fisher). Normalization was carried out according to the manufacturer’s default protocol converting probe cell intensity (CEL files) into signal data (CHP files).

Splice variant analysis was performed using the Gene + Exon function in TAC which uses a gene normalized intensity value (ratio of probe set intensity to gene expression level).

**Supplementary File 1: Supplementary figures.**

**Supplementary Figure 1: Differentially expressed DRG neuron genes.** Differentially expressed genes in ( **A**) Bone cancer, ( **B**) PSL, ( **C**) MJL, ( **D**) Oxaliplatin, ( **E**) CMP and ( **F**) CFA with known basal DRG neuron expression in the Usoskin *et al.* dataset ^4^. Genes listed here correlate with those shown in Figure 7. Analysis has been restricted to DEGs with a fold-change greater than 1.5 or less than -1.5 (p<0.05) with at least 20% expression in 1 or more of the neuronal subgroups in the Usoskin *et al.* dataset ^4^. Genes with an asterisk are present within the *Pain Genes Database* of pain-related transgenic knockout studies ^34^.

**Supplementary Figure 2: Differentially expressed microRNA genes.** Differentially expressed microRNA DRG genes in ( **A**) Bone cancer, ( **B**) PSL, ( **C**) MJL, ( **D**) Oxaliplatin, ( **E**) CMP and ( **F**) CFA pain models (fold change >1.5, <-1.5).

**Supplementary Figure 3: Differential splicing analyses.** Top 10 splice variants for ( **A**) Bone Cancer, ( **B**) PSL, ( **C**) MJL, ( **D**) Oxaliplatin, ( **E**) Chronic Muscle Pain and ( **F**) CFA. Attached heat map for each model shows the splicing index (SI) of the spliced genes (exon SI >2 or <-2, Exon p-val <0.05). Exon event score calculated by TAC software (see methods) where 1 represents a high likelihood for the spliced event. Exon events were defined in TAC as: Intron retention- when a sequence is spliced out as an intron or remains in the mature mRNA transcript; Cassette Exon (skipped exon) – when one exon is spliced out of the primary transcript together with its flanking introns; Alternative 3`acceptor site when two or more splice sites are recognized at the 5` end of an exon, an alternative 3` splice junction is used changing the 5` boundary of the downstream exon; Alternative 5` donor site when two or more splice sites are recognized at the 3` end of an exon, an alternative 5` splice junction is used changing the 3` boundary of the upstream exon.
