# Supplementary figures and images for: Distinct transcriptional responses of mouse sensory neurons in models of human chronic pain conditions
Source: Wellcome Open Res. 2018 Jun 25;3:78. [Version 1] doi: 10.12688/wellcomeopenres.14641.1 (PMC6053702; doi:10.12688/wellcomeopenres.14641.1)

Supplementary Fig. 1

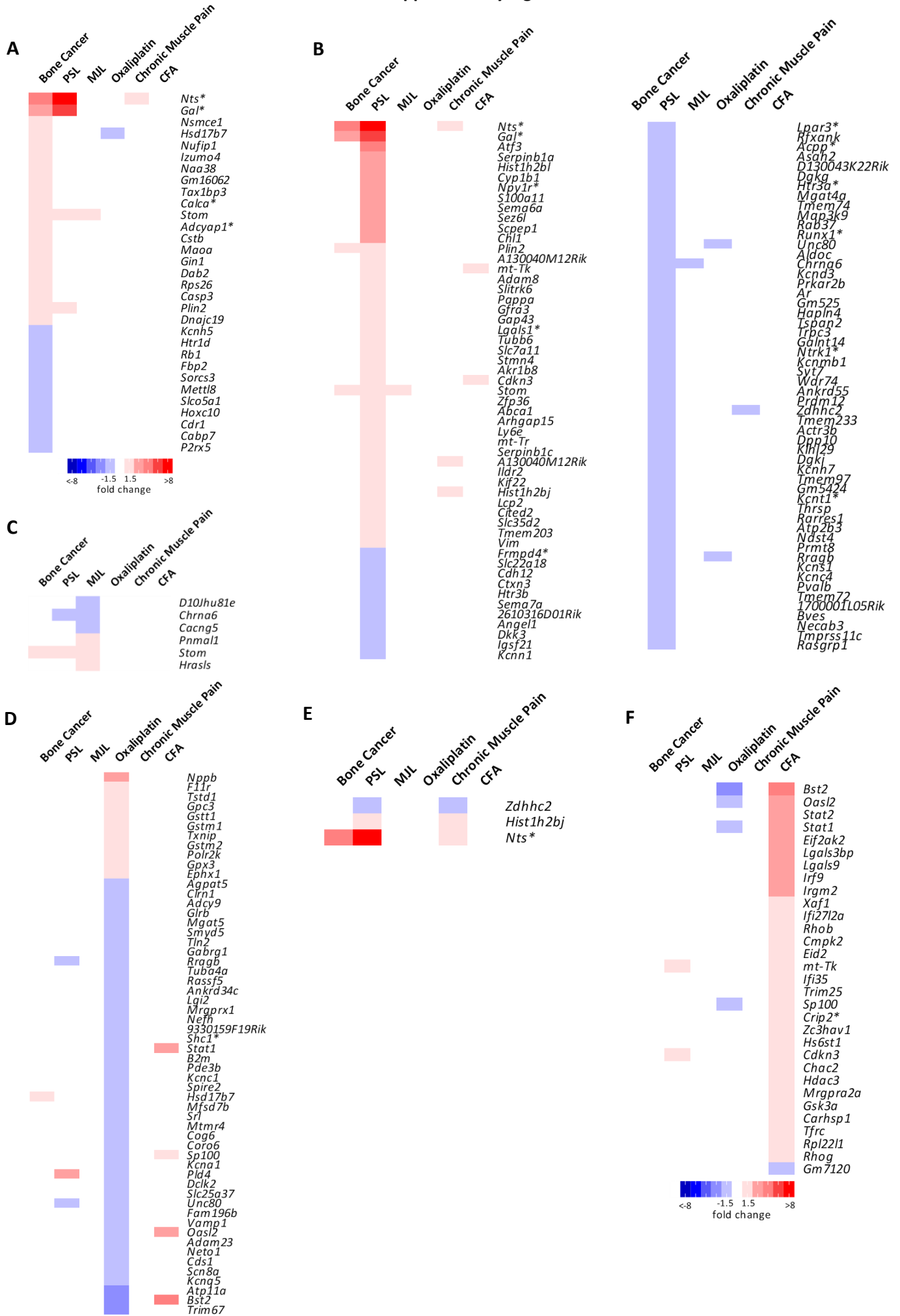

Supplementary Fig. 2

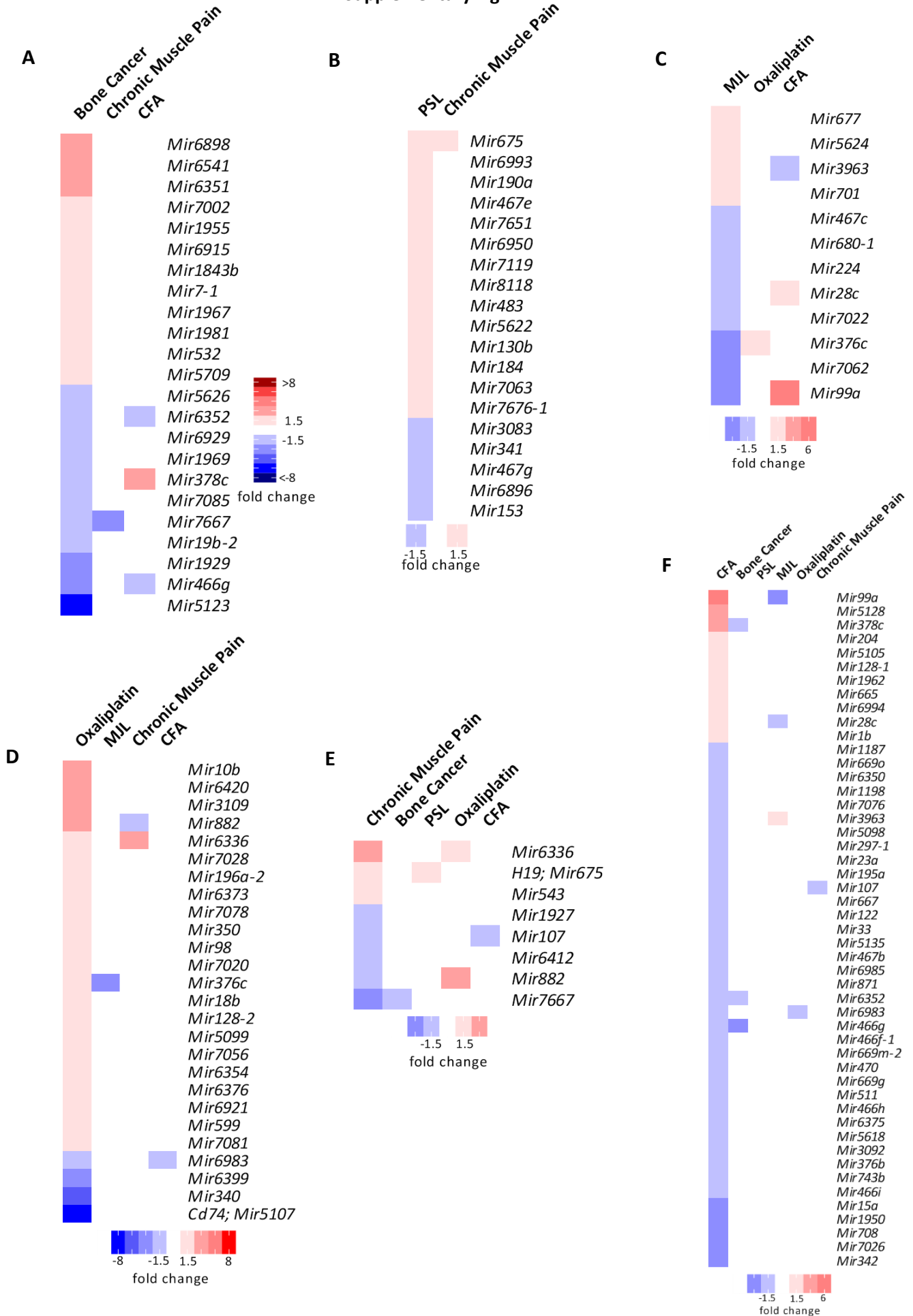

Supplementary Fig. 3

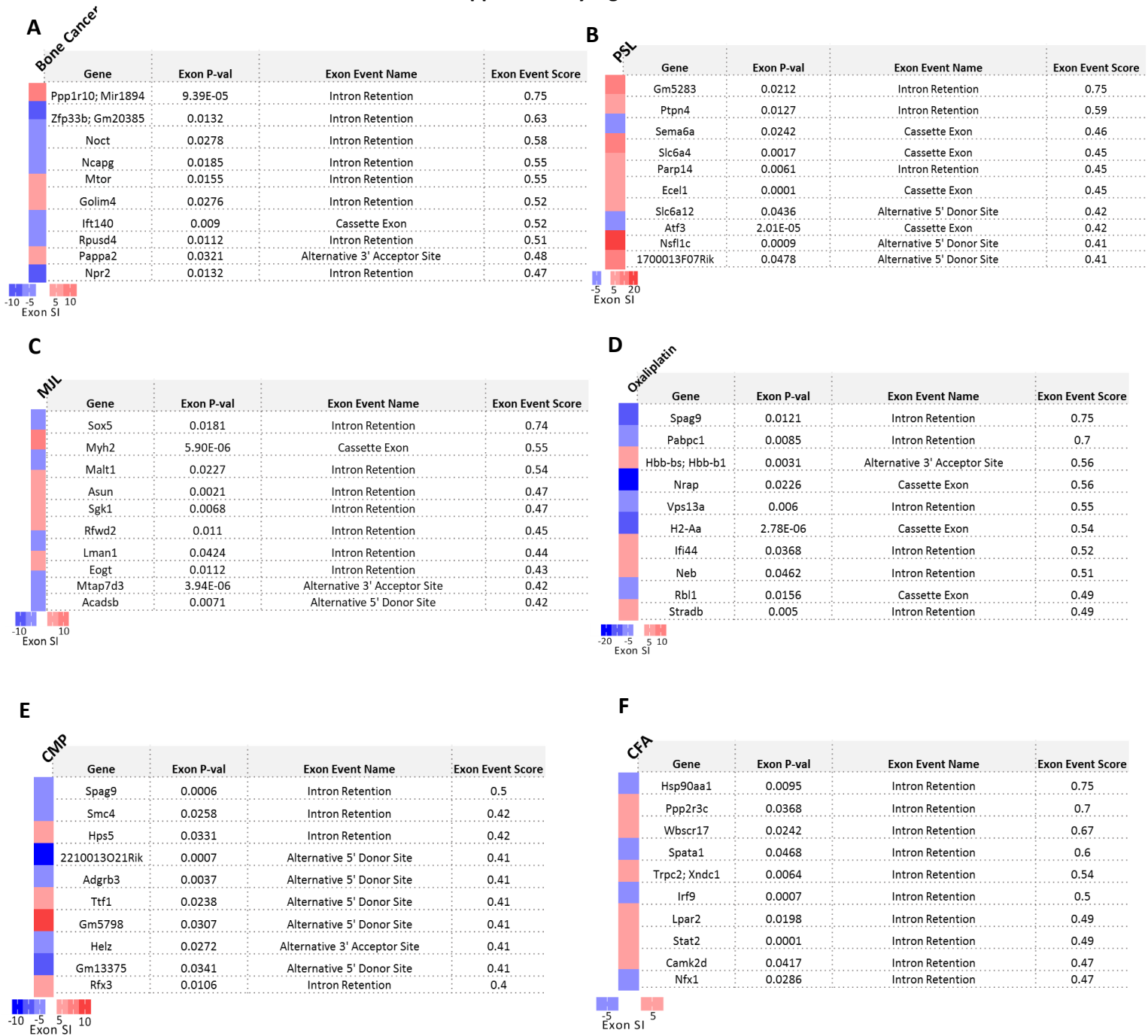

Supplement: Supplementary file 2 [file wellcomeopenres-3-15942-s0001.tgz › 3aa76c1a-87cc-46a7-9301-5fa8f2605bc4.pdf]
