# Supplementary material for: Distinct transcriptional responses of mouse sensory neurons in models of human chronic pain conditions
Source: Wellcome Open Res. 2018 Jun 25;3:78. [Version 1] doi: 10.12688/wellcomeopenres.14641.1 (PMC6053702; doi:10.12688/wellcomeopenres.14641.1)
Supplement: Peer review history

# Peer review: Referee response for version 1

**DOI:** 10.21956/wellcomeopenres.15942.r33489

**Published:** 2018-07-18

**Authors:** Mark D. Baker

**Affiliations:** 1 Blizard Institute, Queen Mary University of London, Whitechapel, UK

The paper discovers differentially expressed genes in mouse primary sensory neurons (DRG) in a variety of pain models that are believed to be models of  human pain states. Looking in the primary neurons the authors find that, although pain levels appear similar and significant in all the models studied, the changes in gene expression for each of the pain states is different. The paper is of great interest, and I have enjoyed trying to grapple with its contents. This is a way of saying that I like it, but I wanted to clarify a couple of things, that have raised a flag.

 The discussion is written as though no gene expression data have ever been obtained before in pain models, and I agree with David Wynick that a more helpful approach would require that some context is offered in the discussion, particularly as this group of authors are generating a benchmark. For example, with reference to the findings on local inflammation, in Figure 2D I do not see a single sodium channel gene affected, and yet Na+ channel genes are reported by others to be dysregulated, for example NaV1.3 expression is reported to double in DRG in inflammatory circumstances ^1^. Do the authors think this is surprising and what is the reader to make of this? In fact, is Na+ channel gene expression in DRG neurons affected at all by any of the induced pain states? What has happened to other potentially important pain related ion channels such as KCNQ and HCN channels?

 Is one correct deduction then that while there may indeed be stereotyped gene expression changes taking place (given that somehow the maladaptive changes in the nervous system exist for all these pain scenarios) they must be in second and higher order neurons, involving glutamate gated ion channel subunits and so on?

 Secondly, as it seems inescapable to conclude that the membrane behaviour of primary sensory neurons must be somehow ‘abnormal’ in pain states driven from the periphery, is the correct conclusion that this is precipitated without any necessity of modification of ion channel expression, completely by a combination of already latent functionality and post-translational modification.

 I would like a little more clarification on Figure 7. I think the authors are looking a for a pattern of gene dysregulation that might conspicuously ‘fit’ the already discovered baseline gene expression profiles of sub-populations of neurons. I think I understand that fitting the profiles against each subtype (previously described) reveals nothing useful, but I would be happy with a bit more explanation for this (bottom page 10 and Figure 7), as I want to rid myself of (I think !) the erroneous impression that single cell analysis is going on.

I have read this submission. I believe that I have an appropriate level of expertise to confirm that it is of an acceptable scientific standard.

## References

[1] BlackJALiuSTanakaMCumminsTRWaxmanSG: Changes in the expression of tetrodotoxin-sensitive sodium channels within dorsal root ganglia neurons in inflammatory pain. *Pain*.2004;108(3) : 10.1016/j.pain.2003.12.035 237-47 10.1016/j.pain.2003.12.035 15030943

# Peer review: Referee response for version 1

**DOI:** 10.21956/wellcomeopenres.15942.r33470

**Published:** 2018-07-13

**Authors:** David Wynick

**Affiliations:** 1 Department of Translational Health Sciences, Bristol Medical School, University of Bristol, Bristol, UK

This is a well written paper that is clearly and accurately presented. The study design is appropriate and the work is technically sound. The data substantially expands the current evidence-base in this field.

 The authors have used transcriptional profiling to identify differentially expressed genes in the mouse DRG following six different chronic pain models. Most interestingly and unexpectedly, they found almost no similarity between the transcriptional changes in expression with very little overlap in the genetic signatures between the models.

 Points that could be further expanded upon in the discussion:

- Place the current dataset in the context of the previous published studies that have used transcriptional profiling on the DRG after different pain models (and in some cases comparing two different models), and whether the previous findings and genes identified are consistent with the current data.
- Identifying genes in the DRG on the basis of their fold-change after a pain model does not mean that those genes are necessarily involved in mediating pain. Indeed, many of the targets of the current analgesics used in clinical practice do not show >two-fold change in expression in mouse models. It is quite possible that many of the genes that do show large changes in each model play roles that relate to apoptosis, survival and/or regeneration, rather than pain transmission and nociception.
- The main finding of this paper provides further evidence that it may not be possible to identify targets in the final common nociceptive pathway that will target all causes of pain and would be amendable to drug discovery. A more targeted and mechanistic approach is likely to be necessary to focus more on specific pathways that are activated and thus on the individual diseases that cause the pain.

I have read this submission. I believe that I have an appropriate level of expertise to confirm that it is of an acceptable scientific standard.
